# Heath-related quality of life in Spanish breast cancer patients: a systematic review

**DOI:** 10.1186/1477-7525-9-3

**Published:** 2011-01-14

**Authors:** María Concepción Delgado-Sanz, María José García-Mendizábal, Marina Pollán, Maria João Forjaz, Gonzalo López-Abente, Nuria Aragonés, Beatriz Pérez-Gómez

**Affiliations:** 1 Department of Cancer and Environmental Epidemiology, National Centre for Epidemiology, Carlos III Institute of Health, Avda. Monforte de Lemos 5, 28029 Madrid, Spain; 2 Consortium for Biomedical Research in Epidemiology & Public Health (CIBER en Epidemiología y Salud Pública - CIBERESP; 3 National School of Public Health, Carlos III Institute of Health, Avda. Monforte de Lemos 5, 28029 Madrid, Spain; 4 Consortium for Biomedical Research in Neurodegenerative Diseases (CIBER en Enfermedades Neurodegenerativas - CIBERNED

## Abstract

**Background:**

Breast cancer is one of the oncological diseases in which health-related quality of life (HRQL) has been most studied. This is mainly due to its high incidence and survival. This paper seeks to: review published research into HRQL among women with breast cancer in Spain; analyse the characteristics of these studies; and describe the instruments used and main results reported.

**Methods:**

The databases consulted were MEDLINE, EMBASE, PsycINFO, Dialnet, IBECS, CUIDEN, ISOC and LILACS. The inclusion criteria required studies to: 1) include Spanish patients, and a breakdown of results where other types of tumours and/or women from other countries were also included; and, 2) furnish original data and measure HRQL using a purpose-designed questionnaire. The methodological quality of studies was assessed.

**Results:**

Spain ranked midway in the European Union in terms of the number of studies conducted on the HRQL of breast cancer patients. Of the total of 133 papers published from 1993 to 2009, 25 met the inclusion criteria. Among them, only 12 were considered as having good or excellent quality. A total of 2236 women participated in the studies analysed. In descending order of frequency, the questionnaires used were the EORTC, FACT-B, QL-CA-Afex, SF-12, FLIC, RSCL and CCV. Five papers focused on validation or adaptation of questionnaires. Most papers examined HRQL in terms of type of treatment. Few differences were detected by type of chemotherapy, with the single exception of worse results among younger women treated with radiotherapy. In the short term, better results were reported for all HRQL components by women undergoing conservative rather than radical surgery. Presence of lymphedema was associated with worse HRQL. Three studies assessed differences in HRQL by patients' psychological traits. Psychosocial disorder and level of depression and anxiety, regardless of treatment or disease stage, worsened HRQL. In addition, there was a positive effect among patients who reported having a "fighting spirit" and using "denial" as a defence mechanism. One study found that breast cancer patients scored worse than did healthy women on almost all SF-12 scales.

**Conclusion:**

Research into health-related quality of life of breast-cancer patients is a little developed field in Spain.

## Background

Taking both sexes into account, breast cancer is the most frequent tumour in Europe [1]. It is one of the oncological diseases for which it has become almost standard practice to include the study of the disease's impact on health-related quality of life (HRQL) among the priority research goals [2], particularly in the English-speaking world [3].

HRQL can be defined as self-perceived aspects of wellbeing that are related to or affected by the presence of a disease or treatment [4]. As a multidimensional construct, it includes perceptions, both positive and negative, of several dimensions such as physical, emotional, social and cognitive functioning. It also includes the negative aspects of somatisation disorder and symptoms caused by a disease and/or its treatment [5]. Studies undertaken in different settings or in different countries might display slight divergences, as HRQL is also modulated by cultural and care patterns.

In the case of breast cancer, disease severity and type of treatment (surgery, radiotherapy, chemotherapy, or a combination of all three) have a clear influence on the patient's subjective perception of the disease. Their effects on HRQL are also modulated by personality traits, personal resources, availability and perception of social and family support [6], as well as by the strong cultural association between the breast and women's self-esteem and sexuality [7].

A recent review of definitions and conceptual models of HRQL applied to oncological patients classifies HRQL-measurement instruments validated for use in cancer patients into two categories. Questionnaires specifically designed for the disease explore the repercussions of the most usual symptoms and side-effects, and are appropriate for comparing treatments or changes in patients. The general instruments are applicable to any population, and are better suited to studies that seek to ascertain the disease's repercussion on HRQL, taking the general population as reference [8]. Among the former, the most used in Europe for breast cancer are the European Organization for Research and Treatment of Cancer Core Cancer Quality Life Questionnaire (EORTC QLQ-C30) and its breast-cancer-specific module (EORTC-BR23). Among the latter, the Medical Outcomes Survey Short-Form General Health Survey (SF-36) is the most widely used [9].

Although breast cancer incidence is lower in Spain than in other European countries, the number of new cases, which was estimated at 22985 women in 2006 [1], has shown a clearly rising trend from the 1980s until the year 2000 [10]. Spanish women are estimated to have a 6%-9% lifetime risk of developing this tumour [11]. Screening programmes, along with diagnostic and therapeutic advances, have led to a steady decline in mortality rates since the early 1990s, [12,13], and the estimated survival at five years of diagnosis currently stands at 86% [14]. Hence, as the number of women living with this tumour in Spain progressively rises, the study of HRQL should become a research topic of increasing relevance. Studies on Spanish breast cancer patients might reflect specificities that could help improve and focus care in such women. To this end, we performed a systematic review of HRQL research targeting breast cancer patients in Spain, analysing the studies published and instruments used, and summarizing the main results reported.

## Methods

### Search strategy

The databases consulted were MEDLINE via PubMed, EMBASE, PsycINFO, Dialnet, *Índice Bibliográfico Español en Ciencias de la Salud *(IBECS), CUIDEN, ISOC and *Literatura Latinoamericana y del Caribe en Ciencias de la Salud *(LILACS). The information found was supplemented by manual searches based on the references cited in the papers initially identified. The MeSH terms used for the search were as follows: "Quality of life" AND "Breast Neoplasm" AND "Spain" in PubMed, EMBASE and PsycINFO, with "Breast Cancer" also being used as a key word; "Quality of life" AND "Breast cancer" in Dialnet; "quality" AND "life" AND "cancer" AND "breast" in IBECS; "quality of life" and "breast cancer" and "Spain" as the key words in CUIDEN; "cancer" AND "breast" AND "quality" AND "life" in ISOC; and lastly, the terms "Neoplasms of breast", "Spain" and "Quality of life" in LILACS.

The search was conducted until August 2009, without restrictions of language or year of publication. In addition, the same search strategy used in the two main databases (PubMed and PsycINFO) was then applied to each European Union (EU) country. This enabled us to obtain an idea of Spain's relative interest in HRQL research in breast cancer patients within a more international context. The systematic search and review processes were conducted in accordance with PRISMA (Preferred Reporting Items for Systematic Reviews and Meta-Analyses) statement criteria.

### Inclusion and exclusion criteria

Detailed analyses were solely performed in the case of papers reporting Spanish studies. Several inclusion criteria were established. In the case of original papers, studies were required: 1) to include Spanish patients with breast cancer; and 2) to furnish original data and measure HRQL as an independent construct, using a specific questionnaire. We excluded any study in which the results were not broken down by country of origin when women from different countries took part, or by type of tumour when patients with different tumour sites were included.

### Data-extraction

Two researchers participated independently and sequentially in the search for and selection of papers. The complete text of the most relevant papers was obtained and the papers were checked for inclusion criteria. Data on the authors' names, year of publication, journal, study title, questionnaire used to measure HRQL, disease stage, type of treatment, sample size, and conclusions about HRQL were collected using a pre-established structured form set out in the review protocol. The information extracted by the two reviewers was then compared and, in case of disagreement, the opinion of a third reviewer was accepted as the criterion for the paper being included or not.

### Quality assessment

The methodological quality of each of the selected papers was assessed through two checklists based on the ones used in two systematic reviews[15,16]. Validation studies were assessed using the following criteria: reliability, type of criterion measure, and validity. Each criterion was rated as high, moderate, or low. These three ratings were condensed into a five-level overall judgment (excellent, good, moderate, fair, or poor) using the same decision rules as Vodermaier et al.[16].

The remaining papers were assessed according to the adapted list of Mols and Denollet's [15] criteria, which included the following items: 1) a validated questionnaire is used; 2) a description is included of at least the stage and type of treatment; 3) inclusion and/or exclusion criteria are described; 4) a information is given about the degree of the sample selection; 5) a participation rates for patient groups are described and are more than 75%; 6) study size of at least 100 participants; 7) the process of data collection is described; 8) the results are compared between two groups or more; and 9) statistical proof of the findings is reported. Each item received a score of one or zero depending on whether or not it fulfilled the criterion. According to the score obtained, studies were arbitrarily considered as: excellent (a score of 8-9); good (6-7); moderate (4-5); fair (2-3); and poor (0-1).

## Results

**Figure 1:**
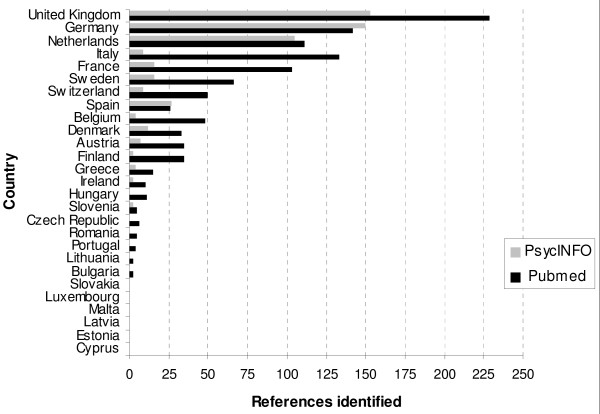
**Results of the literature search made for each European Union country in PubMed and PsycINFO**. References identified in the PubMed and PsycINFO databases in European Union countries, using "Breast cancer", "Quality of life" and country name as descriptors.

Figure 1 graphically depicts the results of the search made for each EU country in PubMed and PsycINFO. While the United Kingdom and Germany were the two countries with most references in both databases, Eastern European countries generally had very few studies. Spain occupied a middle-ranking position according to PubMed, although it was one of the countries with most references in PsycINFO.

**Figure 2:**
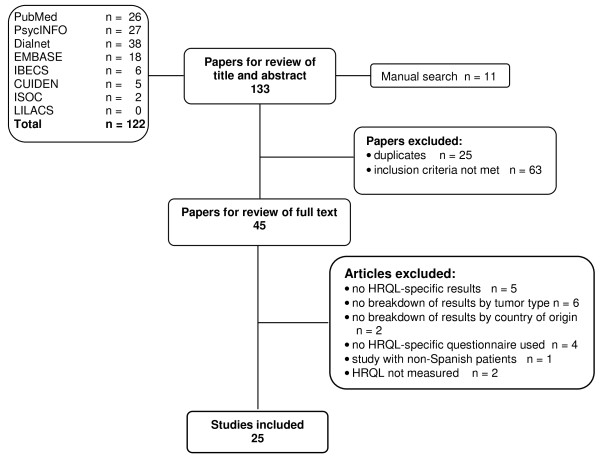
**Flow chart of process of systematic literature search**. Flow chart of systematic search and review process conducted in accordance with PRISMA (Preferred Reporting Items for Systematic Reviews and Meta-Analyses) statement criteria.

Figure 2 shows the flowchart of the systematic review process followed in the case of Spanish papers. The initial search located a total of 133 publications, 82% in Dialnet, PsycINFO, PubMed and EMBASE. After duplicated references had been discarded, 45 papers were reviewed, with 25 papers that fulfilled the inclusion criteria being identified. The studies selected are shown in Table 1. All were published from 1993 to 2009, with the majority (80%) being published in home-based Spanish-language journals and only five in international English-language journals. A total of 2236 women participated in these studies, with sample sizes ranging from 10 to 583 participants (median, 98). The table included as Additional File 1 lists the basic characteristics of the studies identified in the review and provides a summary of their principal results.

**Table 1:** Studies on quality of life and breast cancer among Spanish women (1993-2009)

Author(s) [Ref.]	Year	Objective	HRQL assessment
Toledo et al. [17]	1993	Adaptation of EORTC questionnaire	EORTC (adapted)

Font [18]	1994	Validation of questionnaire	QL-CA-Afex

Ferrero et al. [38]	1994	To explore the relationship between mental adaptation to cancer and QL	EORTC (adapted)

Ferrero et al. [39]	1995	Time trend in HRQL and its association with coping with the disease	EORTC (adapted)

Blasco et al. [29]	1995	To ascertain the effect on HRQL of autologous treatment with support of peripheral hematopoietic cells	EORTC (adapted) QL-CA-Afex

Cagigal Rodríguez et al. [30]	1995	Comparison of the effect of 3 different types of chemotherapy on HRQL	RSCL FLIC

Sprangers et al. [19]	1996	Validation of questionnaire	QLQ-BR23

Toledo et al. [32]	1996	To assess global QL after surgery and before adjuvant treatments	EORTC (adapted)

Ruiz et al. [22]	1998	To establish a psychological profile for interventions targeted at improving psychosocial adjustment	CCV

Sebastián et al. [23]	1999	To assess the efficacy of a psychological group intervention program	EORTC (adapted)

Condón et al. [36]	2000	To ascertain the impact of lymphedema on QL	QLQ-C30 QLQ-BR23

Arraras et al. [20]	2000	Psychometric study of the QLQ-C30 questionnaire (version 2.0)	QLQ-C30

Arraras et al. [21]	2001	Validation of the QLQ-BR23 questionnaire with Spanish sample	QLQ-B23

Arraras et al. [33]	2001	To assess QL during treatment	QLQ-C30 QLQ-B23

Arraras et al. [34]	2003	To assess QL after a long follow-up period, and study differences in QL by disease stage, surgery, and adjuvant treatment	QLQ-C30 QLQ-BR23

Font et al. [24]	2004	To show the efficacy of a psychological group intervention, by analyzing variations in HRQL before and after therapy	QL-CA-Afex

Cervera et al. [41]	2005	To study the impact of diagnosis of breast cancer on patients' psychological (psychopathology and QL) and conjugal adjustment	SF-12

Manos et al. [40]	2005	To analyze the influence of some variables (socio-demographic, type of surgery, social support, and QL) on body image and self-esteem	EORTC (adapted)

Sánchez et al. [25]	2005	To analyze variables related with withdrawal from a psychological group intervention program	QLQ-C30

Herrero et al. [35]	2006	To evaluate the efficacy of a cardiovascular training and resistance program vis-à-vis functional capacity and QL	QLQ-C30

Yélamos et al. [37]	2007	To compare the QL of women operated on breast cancer with and without lymphedema	FACT-B +4

Páez et al. [26]	2007	To compare acceptance and commitment therapy as against cognitive-behavioural therapy.	FACT-B

Bellver [27]	2007	To assess the efficacy of two types of group therapy on the emotional state and QL of women with breast cancer, on termination of adjuvant treatments.	FACT-B

Arraras et al. [31]	2008	To make a prospective assessment of the QL of elderly patients who initiated treatment with radiotherapy, and compare it with that of a sample of younger patients	QLQ-C30 QLQ-BR23

Manos [28]	2009	To assess a psychosocial intervention program	EORTC (adapted)

EORTC: European Organization for Research and Treatment of Cancer; HRQL: health-related quality of life; QL: quality of life

### Study objectives

The goal of five studies was the validation or adaptation of questionnaires [17-21]. Another fourteen papers focused on studying the repercussions of different therapies on patients' HRQL. The most common objective (seven studies) was assessing the possible beneficial effect of psychotherapy and related factors [22-28]. Insofar as medical treatments were concerned, the studies compared the effects on HRQL of different types of chemotherapy [29,30], radiotherapy [31] or surgery [32-34]. Similarly, one pilot study examined the efficacy of cardiovascular training on HRQL [35]. In addition, two papers investigated how HRQL might be influenced by lymphedema [36,37]. Two research studies considered variability in patients' HRQL according to personality traits [38,39]. Another analysed the influence of HRQL on body image and self-esteem [40]. Lastly, one study addressed the overall repercussions of the disease, comparing the HRQL of women with breast cancer with that of healthy women [41].

### Questionnaires

Seven HRQL instruments were identified. The EORTC questionnaires were the most used, in both the original form QLQ-C30 and the Spanish version, with a specific module for breast cancer, i.e., the QLQ-BR23 [19-21,25,31,33-36], and the version adapted for breast cancer by Toledo in 1993 [17,23,28,29,32,38-40]. Some of the retrieved studies made express reference to the construction and validation of the breast-specific module [19]. The QLQ-C30 [42] is a self-administered questionnaire specifically for cancer patients, drawn up and designed to be used in national and international studies. It is relatively short, with a mean completion time of 10 minutes. It has a multidimensional structure, with multi-item scales that cover four basic HRQL dimensions: disease symptoms and toxicity; physical functioning and role-physical; psychological dimension; and social dimension. It uses Likert-type scales, and all dimensions are summarized in a final score, ranging from 0 to 100. In Spain, this questionnaire has been the subject of validation studies, using samples of patients with cancer of the breast, lung, and head and neck [20,43].

The breast-cancer-specific QLQ-BR23 questionnaire [19] assesses areas related to the various treatment modalities (surgery, radiotherapy, chemotherapy, and hormonal therapy). It also assesses other specific aspects of HRQL in breast cancer patients, such as body image or sexuality. The questionnaire consists of two parts: level of functioning and symptoms, each with several individual scales and items. One of the studies included here [17] reported the validation of this questionnaire with a Spanish population. While Toledo's questionnaire [17] can be considered as having good construct validity and internal consistency, neither the version of the EORTC questionnaire on which it was based, nor the process of translation, is clearly specified.

Three studies published in 2007 [26,27,37] used the Functional Assessment of Cancer Therapy-Breast (FACT-B) quality of life instrument, a questionnaire made up of 36 items [44] and designed to measure the HRQL of breast cancer patients in different dimensions. Like the EORTC questionnaires, the FACT-B comprises a general instrument for cancer, FACT-G, complemented by a subscale containing items specific to breast cancer. The items are grouped into five subscales and are answered through five-point response scales. The FACT-B displays good reliability, validity and sensitivity to change [45].

A third instrument for measuring the HRQL in breast cancer patients was the QL-CA-Afex, created by Antóni Font in 1988 and used by the author in two studies, one of which was a validation study [18,24]. The QL-CA-Afex comprises 27 items (visual analogue scales) scored from 0 to 100 and grouped into the following 4 subscales: symptoms; autonomy; familial and social difficulties; and psychological difficulties. Although it is an instrument for chronic diseases, it has been validated with breast cancer patients. One of the studies, with a sample size of only 10 patients, showed a low correlation coefficient with the EORTC instruments [29].

Only one study [30] used the Functional Living Index Cancer (FLIC), a general HRQL questionnaire for cancer patients. It evaluates five HRQL dimensions: physical wellbeing; emotional status; sociability; family situation; and side-effects of treatment [46]. The FLIC displays good content validity and is easy to administer. We failed to find a version of this questionnaire adapted to and validated for the Spanish population. This same study [30] also included the version of the Rotterdam Symptom Checklist (RSCL) geared to the breast cancer population. The applied version has 38 items using a 4-point Likert-type response scale. It assesses functional level, symptoms, psychological functioning and sexual relations, and includes a global HRQL score. Like the previous questionnaire, the RSCL version used was neither adapted to nor validated for the Spanish population, since the cultural adaptation dates from 1998 [47] and the study in question was conducted in 1995.

Lastly, two studies used generic HRQL instruments. One [41] used the SF-12, a short version of the SF-36 for the general population. It is a questionnaire which has been translated into and validated in Spanish. The SF-12 is made up of 8 subscales (physical health, physical functioning, bodily pain, general health, vitality, social functioning, emotional functioning, and mental health), summarized into two global measures: physical and mental. Another study [22] used the Quality of Life Questionnaire (*Cuestionario de Calidad de Vida*, *CCV*), originally developed in Spanish and formally validated in Spain [48]. It comprises 39 items grouped into 4 subscales: general satisfaction, social support, physical/psychological wellbeing, and absence of work overload/leisure time.

### HRQL results for women with breast cancer

#### Comparison between patients and healthy women

The only study [41] to address this objective reported that women with breast cancer displayed greater deterioration in their HRQL, scoring worse on almost all SF-12 scales.

#### Treatments and pathology associated with the disease

With respect to the studies about the influence of treatments on HRQL, one [30] reported few differences in HRQL among the study groups receiving different types of chemotherapy. As regards the influence of radiotherapy on the HRQL of persons aged 65 years and over, one study [31] observed no significant differences prior to and at 6 months of treatment.

Insofar as psychotherapy was concerned, one study [24] found better HRQL scores in groups that underwent some type of psychological group therapy than in those that received no treatment. Another study [28] observed less depression and psychological distress among those treated. However, no relationship was found between withdrawal from group psychotherapy and HRQL [25,27]. With respect to the type of psychotherapy, one paper [26] concluded that acceptance and commitment therapy yielded better HRQL results than the cognitive therapy which the control group received.

Studies which assessed the effect of surgery [31,34,41] reported that conservative surgery had fewer repercussions on HRQL than did radical surgery, and that HRQL was better for women with sentinel lymph node biopsy than aggressive axillary surgery. One pilot study [35] observed significant improvements in global quality of life and physical functioning in the group of women who had taken part in a cardiovascular training program versus the control group. The two studies on HRQL and lymphedema [36,37] both concluded that the presence of this disorder was associated with worse HRQL.

#### Psychological traits

The studies that assessed variations in HRQL according to patients' psychological traits observed that psychosocial disorders worsened HRQL. In addition, the patients who best preserved their HRQL used coping strategies suited to the situation, maintained high self-referential outcome expectations, and had a certain subjective sensation of control over the disease and its effects [38]. Similarly, significant differences were observed in quality of life according to the level of depression and anxiety, regardless of treatment or disease stage [22]. One study observed a positive effect on HRQL among patients who reported having a "fighting spirit" and using "denial" as a defence mechanism, whilst "helpless/hopeless", "fatalism" and "anxious preoccupation" were associated with worse HRQL [39].

### Methodological quality of the Studies

The additional file provides the summary judgments for the predefined evaluation criteria. Three of the studies were considered to be of poor quality according to our checklist [17,19,21]. Only one paper was evaluated of a fair quality [29]. Nine were assessed of a moderate quality [18,22,26,30-32,34,38,40]. Of the remaining studies, eleven were of a good quality [20,23-25,27,28,33,35-37,39] and one paper was of an excellent quality [41].

## Discussion

Our results indicate that research into the HRQL of breast cancer patients is a little developed field in Spain. Despite being one of the most populated countries in Western Europe, Spain ranks among those with the lowest number of studies addressing this issue. There appear to be two different publishing patterns among European researchers active in this field. In the majority of cases, papers are published in medical journals, and so PubMed is the bibliographic database with most information. In the case of Germany and The Netherlands, however, there are similar numbers of papers in PsycINFO and PubMed, probably reflecting a more multidisciplinary approach. This would also seem to apply to Spain. Almost half the studies included in this review were indexed in PsycINFO. Fewer papers were published from the medical side. Indeed, the leading medical bibliographic database, PubMed, contained only four of the studies included in this systematic review, all of which had been published in international journals [19,28,31,35].

In this systematic review, we found only 25 papers that met all inclusion criteria. Five of these, published between 1993 and 2001, reported validations or cultural adaptations of questionnaires. Most of the others examined HRQL by type of treatment, or analysed the influence of treatments and specific symptoms on HRQL. A different approach was shown by the three studies that assessed differences in HRQL by psychological traits. Lastly, one report compared the HRQL of patients with healthy women.

The total number of papers identified was low, despite the general recommendation to include HRQL as an outcome in clinical studies involving breast cancer patients [49], and the fact that HRQL questionnaires are routinely used in clinical research. This reflects the scant attention paid to the information afforded by these instruments. Excluding validation studies, the instruments most commonly used to measure quality of life were the EORTC questionnaires (EORTC QLQ-C30 and QLQ-B23) or modified versions of these (13 studies), with the FACT-B ranking second (3 studies). Aside from being instruments with adequate psychometric characteristics for measuring HRQL in cancer patients [19,42,45], they are also the most widely used in international breast cancer studies [3]. However, only the EORTC QLQ-C30 and QLQ-B23 have versions validated in Spanish [20,21,43].

Breast cancer is one of the neoplasms in which the treatment of choice, surgery, tends to be combined with other therapeutic strategies, such as chemo-, radio- or hormonal therapy. Yet, our results show that studies in Spain aimed at evaluating the effect of treatments on HRQL are clearly insufficient, and in the case of some therapies, non-existent. Surgical repercussions on HRQL are a relatively common issue in international studies [3]. This is because surgery is the initial treatment, at least in stages I, II and III, for most breast cancer patients [50]. Nevertheless, there is a notable lack of papers in Spain focused on the effect of surgery on HRQL. Indeed, this review was able to locate only four papers which made reference to this topic [31,32,34,41], and only one of these included the questionnaire scores [41]. This last-mentioned study compared conservative with radical surgery. It reported better results for the former treatment in all components of HRQL in the short term. In this particular instance, differences were not studied by age group, though these were reported in a paper from Canada [51]. Studies undertaken in other countries (Canada, Germany, and Japan) report contradictory results for these two surgical approaches in terms of differences in HRQL in the long term [51-53]. Only one of the Spanish studies considered axillary surgery [31]. While the authors reported that HRQL was better among sentinel lymph node biopsy patients than axillary emptying, they failed to furnish the specific results.

Insofar as radiotherapy was concerned, we found a single follow-up study [31], which observed no significant differences at 6 months of treatment, whether overall or by age group. This reinforces the idea that age should not be the only factor considered when deciding about oncology treatments, as reported in a study from the United Kingdom [54].

The most common objective (seven studies) was to assess the possible beneficial effect of psychotherapy [22-28]. The improvements in HRQL associated with psychological therapies observed in Spanish studies are consistent with the results reported in two papers from the USA and Australia, respectively [55,56]. Two studies about the relationship between mental adaptation to cancer and HRQL observed differences according to patients' age and coping strategies [38,39]. These results partially agree with those yielded by a study that targeted older women from the USA [57]. Nevertheless, it is difficult for our results to be compared with those of other studies, due to differences in study goals and in HRQL assessments involving psychological factors [58,59].

Only two studies addressed the negative impact of lymphedema on the HRQL of Spanish women with breast cancer [36,37], with findings consistent with two USA case-control studies [60,61]. In addition, our review revealed a lack of studies addressing the impact of other common symptoms, such as pain, fatigue or menopausal symptoms. This is in contrast with the attention paid to these items in international literature [3].

Just one study compared the HRQL of breast cancer patients with that of healthy women [41]. This is an area of great interest for quantifying the impact of breast cancer on our society in terms of HRQL. However, no longitudinal study was identified that was capable of evaluating trends in HRQL over time. In view of the low fatality rate of breast cancer patients at the present time, it would be of great value to have access to studies designed to assess the long-term repercussions of the disease on the lives of the women affected.

Some studies (e.g., such as those undertaken by the Breast Cancer Study Group (http://www.ibcsg.org/), in which a Spanish team participated [62], and the papers by Martín et al [63,64]), were excluded in spite of Spanish patients being included in the sample. This was due to failure to report the results with a breakdown by country. Similarly, no consideration was given to studies that pooled patients with different types of cancer but furnished no specific information about women with breast cancer [65-69]. Finally, we also excluded studies that failed to use HRQL-specific instruments [70] or reported no specific results for this construct [71].

The bibliographic search-and-screening phase showed us that the term HRQL has not been interpreted by some Spanish authors in line with the concept of the multidimensional construct to which it refers. According to the WHO, quality of life is, "*an individual's perception of his/her position in life in the context of the culture and value systems in which he/she lives, and in relation to his/her goals, expectations, standards and concerns*" (1994). This definition stresses the importance of self-evaluation of cultural factors [72]. It was for this reason that studies using one-dimensional questionnaires on anxiety, depression or other mental states or physical symptoms, without considering the multidimensionality of the HRQL construct, were excluded from the analysis.

This study, aimed at reviewing research on HRQL among breast cancer patients in Spain, is purely descriptive in nature. The small sample size of existing studies conducted directly in Spain, with almost half the studies identified including fewer than 100 women [23,25,26,29-31,35,36,38-40], renders it difficult to draw conclusions about the HRQL of Spanish breast cancer patients. In such a context, publication bias cannot be regarded as a major problem when compared with the lack of studies providing data on the subject. A further problem was the use of HRQL questionnaires that were in-house and/or not validated for the Spanish population. Lack of information on adaptation and translation of questionnaires is an additional limitation when it comes to comparing, generalizing and, above all, replicating results. The use of questionnaires adapted to and validated for the Spanish population is indispensable for lending credibility to and standardizing the results obtained.

Although there is abundant international research in this field [2,3], the results might not be wholly applicable to our patients, since perception of HRQL is linked to an immediate reality that is specific to the woman who has the disease. Accordingly, our study complements an important review by Montazeri [3], which excluded papers not written in English. The concept of HRQL depends on cultural norms, behaviour patterns, and personal expectations [72]. It presupposes the ability to make a cultural synthesis of all the elements regarded by a given society as making up its pattern of comfort and wellbeing [73]. Moreover, it is reasonable to assume that if there are socio-cultural and health care differences there will also be differences in assessment of HRQL among breast cancer patients in different countries, as has been shown in the case of other tumours [74]. In this respect, attention should be drawn to the fact that international studies do not routinely furnish comparative information on HRQL stratified by country.

This paper is the first one to include articles in Spanish about studies on the quality of life of women with breast cancer, not included in previous reviews. The comprehensive search performed, which included international as well as national bibliographic databases, as well as the review process have been performed following PRISMA statement criteria. However, we could not perform a meta-analysis due to the small number of studies found and to the heterogeneity of goals and results. Additionally, the exclusion of some international studies[62-64] due to the inclusion criteria adopted could be seen as a limitation of the review in its goal to reflect the research made in Spain on HRQL in Breast Cancer patients.

In brief, there is clearly insufficient information available on the HRQL of Spanish women with breast cancer. Since HRQL is one of the principal result indicators for improving the care of and evaluating new treatments for such patients, encouragement should be given to promoting research and publication in this field.

## Abbreviations

CCV: Quality-of-life questionnaire (*Cuestionario de Calidad de Vida*); CUIDEN: Nursing database kept by the Index Foundation; EORTC: European Organization for Research and Treatment of Cancer; EORTC QLQ-BR23: European Organization for Research and Treatment of Cancer Quality Life Questionnaire Breast Cancer Specific Module; EORTC QLQ-C30: European Organization for Research and Treatment of Cancer Core Cancer Quality Life Questionnaire; FACT-B: Functional Assessment of Cancer Therapy-Breast; FLIC: Functional Living Index Cancer; HRQL: Health-related quality of life; IBECS: Spanish Health Science Bibliographic Index (*Índice Bibliográfico Español en Ciencias de la Salud*); ISOC: Social sciences and humanities database kept by the Spanish Research Board (*Consejo Superior de Investigaciones Científicas *- *CSIC*); LILACS: Latin-American & Caribbean Health Science Literature (*LIteratura LAtinoamericana y del Caribe en Ciencias de la Salud*); RSCL: Rotterdam Symptom Checklist; SF-36 & SF-12: Medical Outcomes Survey Short-Form General Health Survey with 36 or 12 items.

## Competing interests

The authors declare that they have no competing interests.

## Authors' contributions

MP and BPG contributed to the conception and design of the review. MCDS and MJGM collected and analyzed the data and drafted the manuscript. MJF and BPG contributed to the analysis and interpretation of the data and to putting the finishing touches to the manuscript. NA, MJF, GLA, MP and BPG conducted a critical review of the manuscript for important intellectual content. All authors had unrestricted access to all data examined and gave their approval to the final version of the manuscript.

## Supplementary Material

Additional file 1 Studies on quality of life and breast cancer among Spanish women (1993-2009): basic characteristics and summary of principal results. Click here for file
