# Supplementary material for: Heath-related quality of life in Spanish breast cancer patients: a systematic review
Source: Health Qual Life Outcomes. 2011 Jan 14;9:3. doi: 10.1186/1477-7525-9-3 (PMC3031190; doi:10.1186/1477-7525-9-3)
Supplement: Additional file 1 — Studies on quality of life and breast cancer among Spanish women (1993-2009): basic characteristics and summary of principal results. [file 1477-7525-9-3-S1.PDF]

Additional file 1. Studies on quality of life and breast cancer among Spanish women (1993-2009): basic characteristics and summary of principal results

Reference	Year	Study population: sample size	Study population: stage	Study population: treatment	Objective	Study design	HRQL assessment	Results	Quality score	Comments
Toledo et al. [17]	1993	n = 179	22 Stage I,<br>126 Stage II<br>31 Stage III	Surgery	Adaptation of EORTC questionnaire	Validation study	EORTC (adapted)	The authors consider that the questionnaire obtained met the psychometric requirements for both construct validity and internal consistency	Poor	Fails to specify clearly what the original questionnaire was, or explain the translation process
Font [18]	1994	n = 35 (pilot study)<br>n = 154 (research to test reliability and validity)<br>n = 215 (prospective study to assess changes over time)	Different stages: diagnosis, treatment, Disease-free interval, recidivated or advanced disease.	Surgery, radiotherapy, chemotherapy, hormonal therapy	Validation of questionnaire.	Validation study	QL-CA-Afex	No assessable data provided	Moderate	Fails to specify the composition of the sample by stage or treatment.<br>Fails to provide structured summary tables
Spranger et al. [19]	1996	n = 496<br>(170 Germans,<br>168 Spaniards and<br>158 Americans)	Stages I-IV	Surgery, radiotherapy, chemotherapy, hormonal therapy or combination	Validation of questionnaire	Validation study	QLQ-BR23	The results lent support to the clinical and transcultural validity of the QLQ-BR23 as a supplementary questionnaire for assessing patients with breast cancer	Poor	
Arraras et al. [20]	2000	n = 177<br>(156 with 2nd measure,<br>141 with 3rd measure)	Various	Surgery with chemotherapy and/or radiotherapy	Psychometric study of the QLQ-C30 questionnaire (version 2.0)	Validation study	QLQ-C30	The statistical performance of the QLQ-C30 questionnaire (version 2.0) was better than that of version 1.0 which it replaced	Good	Same sample used in another two studies
Arraras et al. [21]	2001	n = 177<br>(156 with 2nd measure,<br>141 with 3rd measure)	Various	Surgery with chemotherapy and/or radiotherapy	Validation of the QLQ-BR23 questionnaire with Spanish sample	Validation study	QLQ-BR23	The QLQ-BR23 questionnaire was a reliable instrument and valid for use with a Spanish sample	Poor	Same sample used in another two studies
Ruiz et al. [22]	1998	n = 322	Stages 0-IV	Without treatment, radiotherapy, chemotherapy or in follow-up 1-2 years after surgery	To establish psychological profile for interventions targeted at improving psychosocial adjustment	Cross-sectional study.	CCV	Significant differences in QL by level of depression and situational anxiety, regardless of diagnosis, treatment or disease stage	Moderate	Fails to include questionnaire scores

Reference	Year	Study population: sample size	Study population: stage	Study population: treatment	Objective	Study design	HRQL assessment	Results	Quality score	Comments
Sebastián et al. [23]	1999	n = 29 (17 intervention, 12 controls)	Non-metastatic	Chemotherapy with CMF and/or radiotherapy and/or hormonal therapy	To assess the efficacy of a psychological group intervention program	Case-control study. Measures repeated before and after treatment	EORTC (adapted)	There were significant differences which indicated that, following the intervention, QL improved on 8 of 11 subscales. In the control group, in contrast, improvement in QL was observed on only 1 subscale	Good	Small sample size
Font et al. [24]	2004	n = 142 (69 intervention, 73 controls)	Exclusion criteria: distal recidivated metastasis, presence of two primary tumors or important diseases that might interfere with group therapy	Without active treatment, except for hormonal therapy.	To show the efficacy of a psychological group intervention, by analyzing variations in HRQL before and after therapy	Case-control study. Measures repeated before and after treatment	QL-CA-Afex	Group therapy improved some aspects of QL	Good	Small sample size
Sánchez et al. [25]	2005	n = 62	Stages I and II (recent diagnosis)	Surgery plus radiotherapy or chemotherapy.	To analyze variables related with withdrawal from psychological group intervention program	Cross-sectional study	QLQ-C30	No relationship found between patients' QL and withdrawal from group treatment	Good	Small sample size
Páez et al. [26]	2007	n = 12	Not specified	Surgery, chemotherapy, radiotherapy, brachytherapy	To compare acceptance and commitment therapy as against cognitive-behavioral therapy	Experimental design covering subjects with repeated measures	FACT-B	Acceptance and commitment therapy showed better QL-related results than did cognitive control therapy	Moderate	Small sample size
Bellver [27]	2007	n = 91 (42 self-esteem group, 49 mental state)	Non-metastatic/ Disease-free	On initiating therapy, adjuvant treatments terminated except for hormonal therapy and/or herceptin.	To assess the efficacy of two types of group therapy on the emotional state and QL of women with breast cancer, on termination of adjuvant treatments	Quasi-experimental longitudinal study.	FACT-B	Emotional status and quality of life scores improved in all women.<br>Hardly any differences among types of therapy	Good	Small sample size
Manos [28]	2009	n = 188	Non-metastatic	Chemotherapy and/or radiotherapy and/or hormonal therapy	To assess a psychosocial intervention program	Case-control study. Measures repeated before and after treatment	EORTC (adapted)	The intervention group reported less depression, less psychological distress, fewer socioeconomic problems, and a higher global QL	Good	

Reference	Year	Study population: sample size	Study population: stage	Study population: treatment	Objective	Study design	HRQL assessment	Results	Quality score	Comments
Blasco et al. [29]	1995	n =10	No details given	Conventional chemotherapy	To ascertain the effect on HRQL of autologous treatment with support of peripheral hematopoietic cells	Longitudinal study	EORTC (adapted)<br>QL-CA-Afex	Low degree of correlation among instruments.<br>High standard deviations	Fair	Small sample size,<br>no control group
Cagigal-Rodríguez et al. [30]	1995	n = 24	Stage IV	Previous treatments not specified	Comparison of the effect of 3 different types of chemotherapy on HRQL	Clinical trial	RSCL<br>FLIC	No evidence of significant differences between type of chemotherapy administered and HRQL dimensions assessed by women	Moderate	Small sample size<br><br>Questionnaires used were neither adapted nor validated for the Spanish population
Arraras et al. [31]	2008	n = 98<br>(50 patient 40-64 years; 48 patient 65 years and over)	Stages I - III	Radiotherapy with or without previous hormonal therapy	To make a prospective assessment of the QL of elderly patients who initiated treatment with radiotherapy, and compare it with that of a sample of younger patients	Measures repeated before and after treatment	QLQ-C30<br>QLQ-BR23	No significant differences found in the QL of patients aged 65 years and over, prior to and at 6 months of treatment<br><br>With respect to the 40-64 age group, these patients had worse global QL and fewer breast cancer symptoms at 6 months of treatment	Moderate	Fails to include questionnaire scores measured in the 40-64 age group in 1996
Toledo et al. [32]	1996	n = 179	22 Stage I,<br>126 Stage II<br>31 Stage III	Surgery	To assess global QL after surgery and before administration of adjuvant treatments	Cross-sectional study	EORTC (adapted)	Very positive assessment of global QL after surgery	Moderate	Fails to include questionnaire scores
Arraras et al. [33]	2001	n = 177<br>(156 with 2nd measure, 141 with 3rd measure)	Various	Surgery with chemotherapy and/or radiotherapy	To assess QL during treatment	Measures repeated before, during and after treatment	QLQ-C30<br>QLQ-B23	The treatments caused some deterioration in QL, which registered improvement after treatment	Good	Same sample used in another two studies<br><br>The Results section reports differences in HRQL by type of surgery, side-effects and disease stage, but furnishes no data

Reference	Year	Study population: sample size	Study population: stage	Study population: treatment	Objective	Study design	HRQL assessment	Results	Quality score	Comments
Arraras et al. [34]	2003	n = 104 (only 46 patients had measures in 1996 and 2001)	Stages I, II and III	Surgery and subsequent radiotherapy, Chemotherapy, hormonal therapy or combinations	To assess QL after a long follow-up period, and study differences in QL by disease stage, surgery, and adjuvant treatment	Longitudinal study. Measures in 1996 and 2001	QLQ-C30 QLQ-BR23	QL was satisfactory and similar in both periods. Differences among types of surgery centered on body image. There were no significant differences in analyses by disease stage or type of treatment	Moderate	Fails to include 1996 questionnaire scores
Herrero et al. [35]	2006	n = 16 (8 intervention, 8 controls)	Stages I and II	Surgery with axillary lymph-adenectomy, radiotherapy and chemotherapy	To evaluate the efficacy of a cardiovascular training and resistance program vis-à-vis functional capacity and QL	Case-control study	QLQ-C30	Significant improvement in global QL and physical function in the intervention versus the control group	Good	Pilot study
Condón et al. [36]	2000	n = 60 (20 with lymphedema, 40 without lymphedema)	Non-metastatic	Without chemotherapy or radiotherapy at date of study	To ascertain the impact of lymphedema on QL	Prospective study. Case-control study	QLQ-C30 QLQ-BR23	Lymphedema is a condition that added deficit to QL among patients treated for breast cancer. This deficit was greater with the passage of time	Good	Small sample size
Yélamos et al. [37]	2007	n = 107 (52 with lymphedema 55 without lymphedema)	Disease-free	Adjuvant treatment terminated (could be receiving hormonal therapy)	To compare the QL of women operated for breast cancer, with and without lymphedema	Case-control study	FACT-B +4	Lymphedema was associated with worse general physical health status, more negative emotional state and worse global QL	Good	Small sample size
Ferrero et al. [38]	1994	n = 68	12 Stage I 42 Stage II 14 Stage III	Surgery plus hormonal therapy or chemotherapy	To explore the relationship between mental adaptation to cancer and QL	Longitudinal study. Measures repeated after surgery, and at 3 and 6 months	EORTC (adapted)	A strong association between mental adjustment to cancer and vague self-reported physical symptoms at the first assessment. Older patients showed higher "Denial" and lower "Anxious preoccupation" scores. "Fighting spirit" and "Denial" were associated with better present and future QL; "Helpless/hopeless", "Anxious preoccupation" and "Fatalism" responses were negatively correlated with well-being	Moderate	Small sample size

Reference	Year	Study population: sample size	Study population: stage	Study population: treatment	Objective	Study design	HRQL assessment	Results	Quality score	Comments
Ferrero-Berlanga et al. [39]	1995	n = 68	12 Stage I<br>42 Stage II<br>14 Stage III	Surgery<br>plus<br>hormonal therapy or chemotherapy	Time trend in HRQL and its association with the disease	Longitudinal study.<br>Measures repeated after surgery, and at 3 and 6 months	EORTC (adapted)	Of the medical variables, only physical symptoms were correlated with future welfare.<br><br>Of the psychological variables, better results were associated with "spirit struggle" and "negation" of the disease, whereas "helplessness", "fatalism" and initial "anxious concern" were associated with discomfort, both present and future	Good	Small sample size
Manos et al. [40]	2005	n = 54	Non-metastatic	Chemotherapy with CMF and/or radiotherapy and/or hormonal therapy.	To analyze the influence of some variables (sociodemographic, type of surgery, social support, and QL) on body image and self-esteem	Cross-sectional study.	EORTC (adapted)	There was a negative correlation between post-surgery deterioration in QL and body image and self-esteem	Moderate	Small sample size
Cervera et al. [41]	2005	n = 583<br>(138 patients recruited immediately after diagnosis,<br>445 healthy women recruited after mammography)	Stage 0 -IIA (86%)<br>Stage IIB (8.8%),<br>Stage IIIA (3.7%),<br>Stage IIIB (1.5%)	Surgery,<br>chemotherapy,<br>radiotherapy.	To study the impact of diagnosis of breast cancer on patients' psychological (psychopathology and QL) and conjugal adjustment	Case-control study.	SF-12	Women with breast cancer displayed more deterioration in almost all aspects of QL and in their conjugal relationships. Those who underwent conservative surgery had better QL than those who underwent radical surgery	Excellent	Only study that included healthy women.

EORTC: European Organization for Research and Treatment of Cancer
HRQL: health-related quality of life
QL: quality of life
